# Multiple Origins of Mutations in the *mdr1* Gene—A Putative Marker of Chloroquine Resistance in *P*. *vivax*

**DOI:** 10.1371/journal.pntd.0004196

**Published:** 2015-11-05

**Authors:** Mette L. Schousboe, Samir Ranjitkar, Rupika S. Rajakaruna, Priyanie H. Amerasinghe, Francisco Morales, Richard Pearce, Rosalyn Ord, Toby Leslie, Mark Rowland, Nahla B. Gadalla, Flemming Konradsen, Ib C. Bygbjerg, Cally Roper, Michael Alifrangis

**Affiliations:** 1 Centre for Medical Parasitology, Institute of Immunology, and Microbiology, University of Copenhagen and Department of Infectious Diseases, Copenhagen University Hospital, Copenhagen, Denmark; 2 Department of Zoology, University of Peradeniya, Sri Lanka; 3 International Water Management Institute, Hyderabad, Andhra Pradesh, India; 4 Universidad San Francisco de Quito, Robles y Pampite, Cumbaya, Ecuador; 5 Faculty of Infectious and Tropical Diseases, London School of Hygiene and Tropical Medicine, London, United Kingdom; 6 Blood-Borne Parasites, Lindsley F. Kimball Research Institute, New York Blood Center, New York, New York, United States of America; 7 Laboratory of Malaria and Vector Research, National Institute of Allergy and Infectious Diseases, National Institutes of Health (NIH), Rockville, Maryland, United States of America; 8 Section of Global Health, Institute of Public Health, University of Copenhagen, Copenhagen, Denmark; Makerere University, UGANDA

## Abstract

**Background:**

Chloroquine combined with primaquine has been the recommended antimalarial treatment of *Plasmodium vivax* malaria infections for six decades but the efficacy of this treatment regimen is threatened by chloroquine resistance (CQR). Single nucleotide polymorphisms (SNPs) in the multidrug resistance gene, *Pvmdr1* are putative determinants of CQR but the extent of their emergence at population level remains to be explored.

**Objective:**

In this study we describe the prevalence of SNPs in the *Pvmdr1* among samples collected in seven *P*. *vivax* endemic countries and we looked for molecular evidence of drug selection by characterising polymorphism at microsatellite (MS) loci flanking the *Pvmdr1* gene.

**Methods:**

We examined the prevalence of SNPs in the *Pvmdr1* gene among 267 samples collected from Pakistan, Afghanistan, Sri Lanka, Nepal, Sudan, São Tomé and Ecuador. We measured and diversity in four microsatellite (MS) markers flanking the *Pvmdr1* gene to look evidence of selection on mutant alleles.

**Results:**

SNP polymorphism in the *Pvmdr1* gene was largely confined to codons T958M, Y976F and F1076L. Only 2.4% of samples were wildtype at all three codons (TYF, n = 5), 13.3% (n = 28) of the samples were single mutant MYF, 63.0% of samples (n = 133) were double mutant MYL, and 21.3% (n = 45) were triple mutant MFL. Clear geographic differences in the prevalence of these *Pvmdr* mutation combinations were observed.

Significant linkage disequilibrium (LD) between *Pvmdr1* and MS alleles was found in populations sampled in Ecuador, Nepal and Sri Lanka, while significant LD between *Pvmdr1* and the combined 4 MS locus haplotype was only seen in Ecuador and Sri Lanka. When combining the 5 loci, high level diversity, measured as expected heterozygosity (H_e_), was seen in the complete sample set (H_e_ = 0.99), while H_e_ estimates for individual loci ranged from 0.00–0.93. Although *Pvmdr1* haplotypes were not consistently associated with specific flanking MS alleles, there was significant differentiation between geographic sites which could indicate directional selection through local drug pressure.

**Conclusions:**

Our observations suggest that *Pvmdr1* mutations emerged independently on multiple occasions even within the same population. In Sri Lanka population analysis at multiple sites showed evidence of local selection and geographical dispersal of *Pvmdr1* mutations between sites.

## Author Summary

Chloroquine combined with primaquine has been the recommended antimalarial treatment for *Plasmodium vivax* malaria infections for sixty years but the efficacy of this treatment regimen is threatened by chloroquine resistance. In this study we describe the prevalence of mutations in the *P*. *vivax* gene, *Pvmdr1* among samples collected in seven endemic countries. The mutations are thought to be associated with chloroquine resistance and here we looked for evidence of drug selection by characterising polymorphism in DNA repeat regions (microsatellite (MS) loci) flanking the *Pvmdr1* gene. Mutations in the *Pvmdr1* gene were mainly identified at codons T958M, Y976F and F1076L. Just 2.4% of samples were wildtype at all three codons, while 63% were single mutants (MYF). Clear geographic differences in the prevalence of these *Pvmdr* mutation combinations were observed. At the flanking MS loci, we found high levels of diversity, and significant differentiation between geographic sites. This pattern of variation could indicate directional selection through local drug pressure. In summary, our observations suggest that *Pvmdr1* mutations and thus, chloroquine resistance has emerged independently on multiple occasions even within the same population.

## Introduction

Malaria is one of the world’s leading causes of mortality and morbidity. Since late in the 1940s the antimalarial drug chloroquine (CQ) has been the primary chemotherapeutic for prophylaxis and treatment of malaria because of its good safety profile, low cost and high efficacy against the blood stages of CQ sensitive (CQS) *Plasmodium* parasites, causing the disease. Since the 1950`s, CQ treatment of *P*. *vivax* infections has been combined with the hypnozoitocidal drug primaquine (PQ) for clearance of the latent *P*. *vivax* liver stages, responsible for later relapses of the disease [1–3]. Compared to *P*. *falciparum*, development of CQ resistance (CQR) in *P*. *vivax* has been relatively slow with the first reports emerging in 1989 in Papua New Guinea (PNG) [4]. Since then, CQR has spread and today it is considered to be present in vivax-malaria endemic countries all over the world (reviewed in [5] and more recently in [6]). Development of CQR has been slower in *P*. *vivax* than *P*. *falciparum* and this is sometimes attributed to the use of combined treatment (CQ with PQ), where PQ acts synergistically with CQ against CQR parasites [7]. It is also proposed that CQR in *P*. *vivax* has a different CQR mechanism than *P*. *falciparum* [8].

Knowledge of the mode of action of CQR in *P*. *vivax* is limited. In *P*. *falciparum*, reduced CQ sensitivity is strongly associated with single nucleotide polymorphisms (SNPs) in the chloroquine resistance transporter-gene, *Pfcrt* [9;10]. However, studies of the *Pfcrt* orthologue in *P*. *vivax*, *Pvcg10*, have not been able to find an association to CQR [8;11]. In *P*. *falciparum* is the P-glycoprotein–like molecule Pgh-1 encoded by *Pfmdr1*, is also associated with CQR though it may only modulate the effects of the *Pfcrt* gene [12;13]. In 2005, Brega *et al*. characterized the *mdr*-like gene *Pvmdr1* in *P*. *vivax* isolates [14], and evidence suggests that SNPs in the *Pvmdr1* gene are a possible genetic determinant of CQR [11;14;15]. Cross-species comparisons led the focus in *P*. *vivax* to be primarily on the *mdr*-codons orthologous to codons implicated in *P*. *falciparum* CQR namely 86, 184, 1034, 1042 and 1246 [14;16]. However codons 91 and 189 which are homologous to codons 86 and 184 in *P*. *falciparum* [14;17] and codons 1071 and 1079 which are homologous to codons 1034 and 1042 in *P*. *falciparum* [14] are rarely polymorphic in *Pvmdr1* Instead SNPs at codons, 976 and 1076, have been detected multiple times [11;14;15;18;19]. Suwanarusk *et al*. observed an association between the Y976F mutation and increased CQ IC_50_ in samples from Thailand and Papua province of Indonesia, and stated that the Y976F mutation is a useful tool to indicate foci of chloroquine resistance [11]. Others detect the mutations, but doubt their association with CQR [15;18;20–23].

Studies of *P*. *falciparum* drug resistance loci have used flanking microsatellite (MS) variation to describe selective sweeps around *Pfdhfr* [24] and *Pfdhps* [25] and *Pfcrt* [26]. This approach has revealed lineages of drug resistance mutant alleles which are derived from a single emergence event. Notably, in *P*. *falciparum* some resistance lineages were found to have spread across vast geographical distances [26;27]. When the same approach was repeated antifolate drug targets in *P*. *vivax Pvdhfr* [28] and *Pvdhps* [29] contrasting results were found. In *Pvdhfr* and *Pvdhps* there was evidence of multiple independent mutation events with little selective sweep around mutant alleles at those loci. This result may reflect the limited antifolate drug selection pressure that has historically been applied to *P*. *vivax*, or it may point to differences in transmission and selection dynamics in the two species. In this study we looked for evidence of drug selection on the CQR candidate *Pvmdr* using MS flanking the *Pvmdr1* gene.

We analysed samples collected from Pakistan, Afghanistan, Sri Lanka, Nepal, Sudan, São Tomé and Ecuador. In Pakistan approximately 83% of the malaria cases are caused by *P*. *vivax* and in Afghanistan, 95% are *P*. *vivax*. In both countries *P*. *vivax* infections are still being treated with CQ + PQ [30]. Around 83% of reported malaria infections in Sri Lanka were caused by *P*. *vivax* and CQ with PQ were still efficient and recommended treatment of *P*. *vivax* infections on the island until autochthonous cases of both *P*. *falciparum* and *P*. *vivax* in Sri Lanka fell to zero [30]. In Nepal 88% of the malaria cases are caused by *P*. *vivax* and treated with CQ + PQ [30]. In Ecuador, *P*. *vivax* accounts for 86% of all malaria infections and is treated with CQ + PQ [30]. To the best of our knowledge no cases of CQR have been reported from either Nepal or Ecuador. Only, 5% of the malaria infections in Sudan are caused by *P*. *vivax* and these are treated with artemether-lumefantrine + PQ [30]. No reports of *P*. *vivax* CQR have been published from Sudan. Falciparum malaria is the main cause of malaria in São Tomé and no recommendations are provided regarding treatment of *P*. *vivax* [30].

The objectives of the present study were to 1) Determine the diversity of SNPs in the *Pvmdr1* gene, a putative marker of CQR, in *P*. *vivax* samples collected from Pakistan, Afghanistan, Sri Lanka, Nepal, Ecuador, Sudan and São Tomé and 2) Characterise flanking MS variation and use this to explore the evolutionary origin of *Pvmdr* mutations.

## Methods

### Sample collection and description of collection sites

The total number of *P*. *vivax* samples analysed in this study was 267. The samples originated from 7 countries: Pakistan (n = 36), Afghanistan (n = 13), Nepal (n = 55), Sri Lanka (n = 136), Ecuador (n = 17), São Tomé (n = 4) and Sudan (n = 6). The samples were all (with the exception of Sao Tomé, and Sudan) derived from larger sets of PCR positive samples and the subset selected by computer randomisation. The DNA from a total of 263 *P*. *vivax* samples selected were already extracted as part of a *P*. *vivax* microsatellite study described previously [31].

*Pakistan and Afghanistan*: Forty-nine samples from a cluster of neighbouring sites in Pakistan and Afghanistan were analysed. Thirty-six were from Pakistan (n = 36); Ashaghroo refugee camp in Adizai from 2003 (n = 10), Adizai Refugee Village in Peshawar from 2004–2005 (n = 3) sampled as part of another study [32] and lastly Adizai, Baghicha and Khagan villages located near Peshawar 2005–2006 (n = 23) described in [33]. Thirteen samples were from Afghanistan collected at the Malaria Reference Center in Jalalabad in 2004–2005 [32]. The samples from all these sites were grouped together because of similar study designs and close geographical distance between the sites.

*Sri Lanka*: The samples from Sri Lanka were collected in 9 malaria endemic districts during 2002–2007, see [34]. For this study, the samples were divided into 9 groups of districts, where after randomised computerisation was used to select samples from each district (*N* = 136).

*Nepal*: Samples from two separate studies in Nepal were grouped together. Thirty-eight samples collected in 2009–2010 from the districts of Jhapa (*N* = 34) and Banke, Chitwan and Dang (*N* = 5) have been previously described in [35]. The other study collected samples in the districts of Kanchanpur (*N* = 5) and Jhapa (*N* = 12) in 2005–2006 as a part of a cross-sectional prevalence survey estimating the malaria burden and risk behaviour in two endemic districts of Nepal (S. Hewitt, personal communication). The Kanchanpur samples were grouped with Banke, Chitwan and Dang.

*Ecuador*: Twenty-one *P*. *vivax* samples were collected from 2007–2009 in the Province of Sucumbíos through the network of laboratories of the Ministry of Health.

*Sudan*: Six *P*. *vivax* samples from Sudan were collected in the village of Asar in Gedaref state in 2006 as a part of an artemether-lumefantrine efficacy trial community based survey [36]. The amount of available *P*. *vivax* DNA was small, and only limited analysis was possible.

*São Tomé*: The island São Tomé is a part of the Democratic Republic of São Tomé and Príncipe in the western equatorial coast of Central Africa. Four samples were available. As with the samples from Sudan, limited analysis was possible because only a small amount of extracted DNA-solution was available.

### Amplification and sequence analysis of the *Pvmdr1* gene

A fragment spanning nucleotide 2596 and 3532 (amino acids 865–1177) of the *Pvmdr1* gene and was amplified using semi-nested primers Pvmdr1-4F [11], Pvmdr1-AS and Pvmdr1-S [14]. Primer sequences are shown in Table 1. Cycling conditions were as follows: 94°C for 15 min, followed by 30 cycles of 94°C for 30 s, 55°C for 1 min, and 72°C for 1 min, and finally 72°C for 10 min. The amplified *Pvmdr1* fragments were sequenced on an ABI Prism 377 (Perkin-Elmer) using the Big Dye terminator reaction mix (Perkin-Elmer). After sequencing, the individual haplotypes were aligned and analysed by use of the DNASTAR-Lasergene software.

**Table 1:** Primers for amplification and sequencing of the *Pvmdr1* gene and four flanking polymorphic repeat regions.

Locus			Primer	Use	Sequence (5’-3’)
*Pvmdr1*			Pvmdr1-4F	1^st^ PCR (F)	CCCTCTACATCTTAGTCATCG
			Pvmdr1-AS	1^st^ and 2^nd^ PCR (R)	ACGTTTGGTCTGGACAAGTATC
			Pvmdr1-S	2^nd^ PCR (F)	ATAGTCATGCCCCAGGATTG
MS	Repeat	Size range			
m9.5	AAT	199–207	m9.5F	1^st^ and 2^nd^ PCR (F)	TATGTGGAGAAGGGGAAACG
			m9.5R	1^st^ PCR, (R)	TCTCGTTATTGCTCGCCACT
			m9.5I-FAM	2^nd^ PCR, (R)	FAM-TGCCACATTCATTCTTGAGC
m10.1	A, AT	266–322	m10.1F	1^st^ and 2^nd^ PCR (F)	GCTGCGCCTATAAAAGTTCG
			m10.1R	1^st^ PCR, (R)	GACTTGGAATTCCCACTGCTA
			m10.1I-FAM	2^nd^ PCR, (R)	FAM-GCACTCCATTGTTTCGACTG
m10.4	GCATTTAT	230–308	m10.4	1^st^ and 2^nd^ PCR (F)	GCAGTACTTTGTTCCTCCAC
			m10.4R	1^st^ PCR, (R)	TCAAACTCAAACGCCTTGC
			m10.4I-FAM	2^nd^ PCR, (R)	FAM-CAAAGGGGGAAATAAAAATTCA
m43.1	TA, GT	419–472	m43.1F	1^st^ and 2^nd^ PCR (F)	GCCATGTAAGCCAGAAGTGC
			m43.1R	1^st^ PCR, (R)	GTTAAAACGAGAAGGCACATAGG
			m43.1-I-FAM	2^nd^ PCR, (R)	FAM-CGTGTGCATACGCAGACATA

(F): forward primer, (R): reverse primer. MS: Microsatellites. FAM: primer labeled with FAM in 5’-end.

### Identification of microsatellites

The *Pvmdr1* gene is located on chromosome 10, and sequences flanking the gene were screened for suitable microsatellite marker loci in the Salvador-1 (Sal-1) reference strain (accessed through the European Bioinformatics Institute homepage (www.ncbi.nlm.nih.gov/). Multiple repeats were identified using the software Tandem Repeats Finder [37] and semi-nested primers designed (Table 1). The primary reaction comprised of 1μl template, 0.5 unit Taq polymerase, 1.1μl Thermopol Reaction buffer (New England Biolabs Inc, Glostrup, Denmark), and 0.4μM dNTPs, 0.1μM of forward (F) and reverse primers (R) with cycling conditions as follows; 2 min at 94°C and then 25 repeated cycles of 30 s at 94°C, 30 s at 42°C, 30 s at 40°C and 40 s at 65°C followed by 2 min at 65°C and a minimum of 10 min at 15°C. In the secondary PCR, the same concentrations of reagents were added, but with 0.15μM of reverse primers (R) and fluorescent-labelled inverse primers (I). The cycling conditions were initiated with 2 min at 94°C followed by 25 repeated cycles of 20 s at 94°C, 20 s at 45°C, 30 s at 65°C, and finished with 2 min at 65°C and 10 min at 15°C.

**Fig 1:**
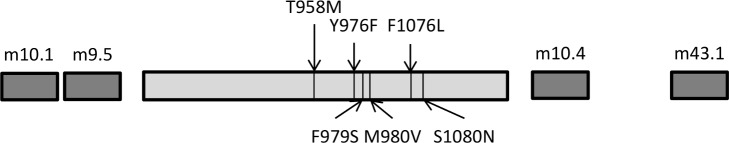
The *Pvmdr1* gene and the relative position of the chosen MS. The identified SNPs within *Pvmdr1* and the two chosen MS downstream of the *Pvmdr1* gene; m9.5 (9,489 bp downstream of *Pvmdr1*), m10.1 (10,120 bp downstream) and the two upstream MS: m10.4 (10,420 upstream) and m43.1 (43,168 bp upstream) are shown.

A panel of four MS were selected for further analysis on the basis of their successful amplification and were named according to their distance to the *Pvmdr1* gene: m9.5 (9,489 bp downstream of *Pvmdr1*), m10.1 (10,120 bp downstream), m10.4 (10,420 upstream) and m43.1 (43,168 bp upstream), (Fig 1). PCR amplified fragments were run with LIZ-500 size standard on an ABI 3730XL genetic analyzer (Applied Biosystems), and analysed using the GeneMapper software (Applied Biosystems). Samples that were negative by PCR were repeat amplified with 2μl template in the first PCR.

The number and length of the repeats in each of the four MS is shown in Table 1. In the cases where multiple (≥ 2) microsatellite alleles were detected in a single sample the major/predominant allele chosen, (‘predominant’ is defined by the electropherogram peak height which had to be twice that of the minor allele).

### Data analysis

Linkage disequilibrium (LD) was calculated to test for a non-random association of *Pvmdr1* allele and the MS alleles. Only the non-mixed samples were used in the calculations. LD was measured by the formula *D' = D/D*_max_, where *D* equals derivation of random association between alleles at different loci, and *D’* measures *D* standardized by the maximum value (*D*_max_), given the observed allele frequencies. LD values range from -1 to +1, where the value +1 refers to a complete non-random association between the alleles. Values of gene diversity were calculated by expected heterozygosity by the formula H_e_ = (*n*/[*n*-*1]*)(*1-∑p*_i_^2^), by use of the Arlequin software [38], where H_e_ is expected heterozygosity, *n* the number of samples, and *p*i the frequency of the *i-*th allele in the sample set.

### Ethical statement

Clearance for analysis of Plasmodium genes were approved by London School of Hygiene Tropical Medicine and Hygiene Ethics Board, locally by Bioethics Committee, Pakistan Medical Research Council and Directorate of Public Health, Jalalabad, Nangahar, Comitee de Bioetica Universidad San Francisco de Quito, Committee on Research and Ethical Review at the Faculty of Medicine, Peradeniya, Kandy and the Nepal Health Research Council. All data analysed were anonymized.

## Results

### Analysis of the *Pvmdr1* gene

**Fig 2:**
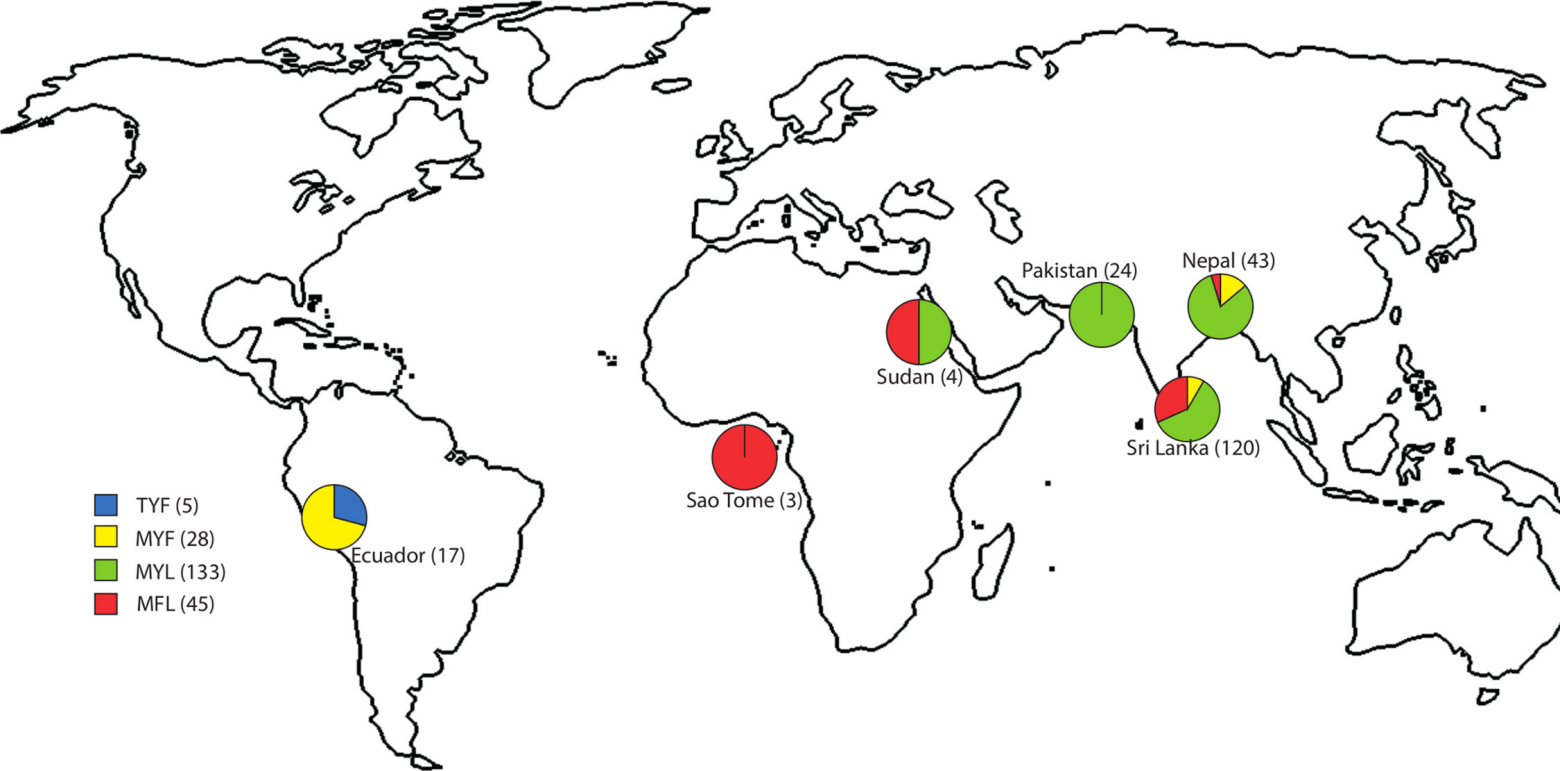
*Pvmdr1* polymorphism in *Plasmodium vivax* samples collected at 8 geographical sites. Four *Pvmdr1* alleles were detected in the study abbreviated as TYF, MYF, MYL and MFL. The TYF wild-type is shown in blue, MYF (single mutant, T958M) in yellow, MYL (double mutant, T958M+F1076L) in green, and MFL (triple mutant T958M+Y976F+F1076L) in red. The number of positive samples from each site is shown on the map in brackets, and the number of alleles and percentage frequency overall are shown in the legend.

The analysis of *Pvmdr1* in 39 samples from Nepal was previously published [35]. Of the remaining 228 samples, sequencing was successful in 173 (75.9%); Pakistan (n = 24), Nepal (n = 4), Sri Lanka (n = 120), São Tomé (n = 3), Sudan (n = 4) and Ecuador (n = 17). Combined with the 39 samples from Nepal, 212 *Pvmdr1* sequence fragments were available for further analysis (Table 2, Fig 2).

**Table 2:** Allelic diversity measured by expected heterozygosity (H_e_).

Origin	*Pvmdr1*	m9.5	m10.1	m10.4	m43.1	5-loci haplotype
Pakistan/Afghanistan	0.00 (1/24)	0.10 (2/20)	0.87 (8/17)	0.74 (6/39)	0.44 (2/29)	0.96 (9/11)
Nepal	0.32 (3/43)	0.56 (4/32)	0.93 (17/37)	0.82 (10/45)	0.40 (3/34)	1.00 (24/24)
Sri Lanka	0.54 (3/120)	0.34 (2/115)	0.82 (12/117)	0.82 (8/122)	0.47 (2/97)	0.97 (39/73)
Ecuador	0.44 (2/17)	0.00 (1/17)	0.68 (4/16)	0.68 (3/16)	0.49 (2/17)	0.83 (8/15)
Sao Tomé	0.00 (1/3)	0.00 (1/2)	1.00 (2/2)	0.00 (1/2)	1.00 (1/1)	—-
Sudan	0.67 (2/4)	0.50 (2/4)	0.50 (2/4)	0.80 (3/5)	0.67 (2/3)	1.00 (1/1)
Mixed infections (%)	—-	2 (1.1)	17 (8.7)	27 (11.8)	22 (12.2)	33 (26.4)
H_e_ (alleles/n)	0.54 (4/211)	0.43 (4/190)	0.90 (26/196)	0.83 (13/229)	0.47 (3/181)	0.99 (78/125)

H_e_ is shown for every locus and for the combined 5 locus haplotype at each study site. In brackets are the number of alleles detected and the sample size.

The number (and percentage) of mixed infections detected at each locus is shown. The 5-loci haplotype calculations included samples with mixed infection and used the majority allele for each locus.

SNP variation in fragment 2 was largely confined to three codons; T958M (ACG→ATG), Y976F (TAC→TTC) and F1076L (TTT→CTT), (Fig 1). Three novel SNPs were detected in two samples from the Jhapa district in Nepal. These were sequenced twice to confirm the results. One of the samples carried a SNP at c1080 (S1080N, AGT→AAT), while the other sample possessed SNPs at c979 (F979S, TTT→TCT) and c980 (M980V, ATG→GTG) (Table 3). These two samples and the SNPs were described by Ranjitkar *et al*. [35].

**Table 3:** *Plasmodium vivax mdr1* alleles TYF, MYF and MYL and flanking microsatellite alleles.

		*Upstream*			*Downstream*		
Origin	5-loci haplotypes number	M43.1	M10.4	Pvmdr	m9.5	m10.1	No. of samples with identical haplotype
Sal-1	—-	441	293	TYF	204	270	**—-**
Ecuador	1^a^	419	261	TYF	201	266	3
Nepal	2	437	266	**M**YF	204	298	1
Ecuador	3	437	266	**M**YF	201	300	1
Ecuador	4	437	266	**M**YF	201	301	1
Ecuador	5	437	266	**M**YF	201	303	1
Sri Lanka	6^a^	419	259	**M**YF	201	283	4
Sri Lanka	7	419	252	**M**YF	201	283	1
Ecuador	8	419	252	**M**YF	201	300	6
Ecuador	9	419	252	**M**YF	201	301	1
Nepal	10	419	233	**M**Y**L**	204	308	1
Sri Lanka	13	419	252	**M**Y**L**	204	282	4
Sri Lanka	14	419	252	**M**Y**L**	204	289	1
Sri Lanka	15	419	252	**M**Y**L**	204	300	1
Nepal	16	419	252	**M**Y**L**	207	289	1
Sri Lanka	17	419	255	**M**Y**L**	204	289	2
Nepal	18	419	259	**M**Y**L**	201	289	1
Nepal	19	419	259	**M**Y**L**	201	266	1
Nepal	20	419	259	**M**Y**L**	201	290	1
Nepal	21	419	259	**M**Y**L**	204	266	1
Nepal^b^	22	419	259	**M**Y**L**	207	277	1
Pakistan	23	419	259	**M**Y**L**	204	280	1
Nepal	24	419	259	**M**Y**L**	204	289	1
Sri Lanka	25	419	259	**M**Y**L**	204	297	1
Sri Lanka	26	419	259	**M**Y**L**	204	298	1
Sri Lanka	27	419	259	**M**Y**L**	204	299	3
Sri Lanka	28	419	259	**M**Y**L**	204	300	1
Nepal	29	419	259	**M**Y**L**	204	300	1
Nepal^c^	29	419	259	**M**Y**L**	204	308	1
Sri Lanka	30	419	266	**M**Y**L**	204	289	4
Sri Lanka	31	419	266	**M**Y**L**	204	298	1
Sri Lanka	32	419	266	**M**Y**L**	204	311	2
Pakistan	33	419	266	**M**Y**L**	204	287	1
Nepal	34	419	266	**M**Y**L**	204	297	1
Nepal	35	419	266	**M**Y**L**	201	315	1
Nepal	36	419	266	**M**Y**L**	201	289	1
Sri Lanka	37	419	270	**M**Y**L**	204	289	4
Sri Lanka	38	419	270	**M**Y**L**	204	299	1
Sudan	39	419	276	**M**Y**L**	207	303	1
Sudan	40	419	308	**M**Y**L**	204	293	1
Sri Lanka	41	437	252	**M**Y**L**	201	297	1
Sri Lanka	42	437	252	**M**Y**L**	204	297	5
Sri Lanka	43	437	255	**M**Y**L**	204	311	1
Pakistan	44	437	255	**M**Y**L**	204	283	1
Sri Lanka	45	437	259	**M**Y**L**	201	289	1
Sri Lanka	46	437	259	**M**Y**L**	204	289	1
Nepal	47	437	259	**M**Y**L**	201	286	1
Sri Lanka	48	437	266	**M**Y**L**	204	303	1
Sri Lanka	49	437	270	**M**Y**L**	204	289	2
Sri Lanka	50	419	244	**MFL**	204	305	1
Sri Lanka	51	419	252	**MFL**	204	283	1
Sri Lanka	52	419	255	**MFL**	201	283	1
Sri Lanka	53	419	255	**MFL**	204	297	1
Sri Lanka	54	419	266	**MFL**	204	299	4
Sri Lanka	55	419	270	**MFL**	204	289	1
Nepal	56	419	276	**MFL**	204	298	1
Sri Lanka	57^a^	437	255	**MFL**	204	291	5
Sri Lanka	58	437	270	**MFL**	204	299	1

The samples from Ecuador (n = 13), Nepal (n = 16), Pakistan (n = 3), Sri Lanka (n = 58) and Sudan (n = 2) a shown together with Sal-1 which is a wild-type chloroquine sensitive haplotype (GenBank accession number AY571984.1). The number of samples per geographical origin, which possessed each distinct haplotype are shown in the last column where there was more than one the row is highlighted dark gray: Excluded in the table are all the polyclonal samples.

^a^5-loci haplotypes calculated to be in significantly linkage disequilibrium (D` = 1, P<0.0001).

^b^A sample from Nepal contained two further mutations at c979 (F979S, TTT→TCT) and c980 (M980V, ATG→GTG).

^c^This sample from Nepal was also mutated at c1080 (S1080N, AGT→AAT).

The substitutions at codons 958,976, and1076 were found in various configurations. TYF (the wild-type) was found only in Ecuador in 5 of 17 samples (Fig 2, S1 Table). All the remaining samples carried one of three mutant haplotypes, MYF (single mutant, T958M), MYL (double mutant, T958M and F1076L) and MFL (triple mutant T958M, Y976F and F1076L). The double mutant MYL was present in 63.0% of the samples (n = 133), the triple mutant MFL in 21.3% (n = 45), and the single mutant MYF in 13.3% (n = 28). Their relative abundance at the different sites is shown in Fig 2. In Pakistan only the MYL double mutant haplotype was detected, while the Ecuador samples (apart from the wild-type TYF) possessed the single mutant MYF (n = 12). The Sudan *P*. *vivax* samples a mix of MYL (n = 2) and MFL (n = 2) haplotypes were found, while the three samples from São Tomé all possessed the MFL haplotype (Fig 2).

The heterozygosity of *Pvmdr1* measured as H_e_ is shown in Table 2. Measured over all samples H_e_ was 0.54. When divided by collection site, Sri Lanka was the most diverse (H_e_ = 0.54), and Pakistan the least diverse (H_e_ = 0) with only one allele-the MYL haplotype. Although the H_e_ value for Sudan was high (0.67), the sample size was small (n = 4), and the broad variation in sample size between sites precluded further in-depth analysis of difference between the sites.

**Fig 3:**
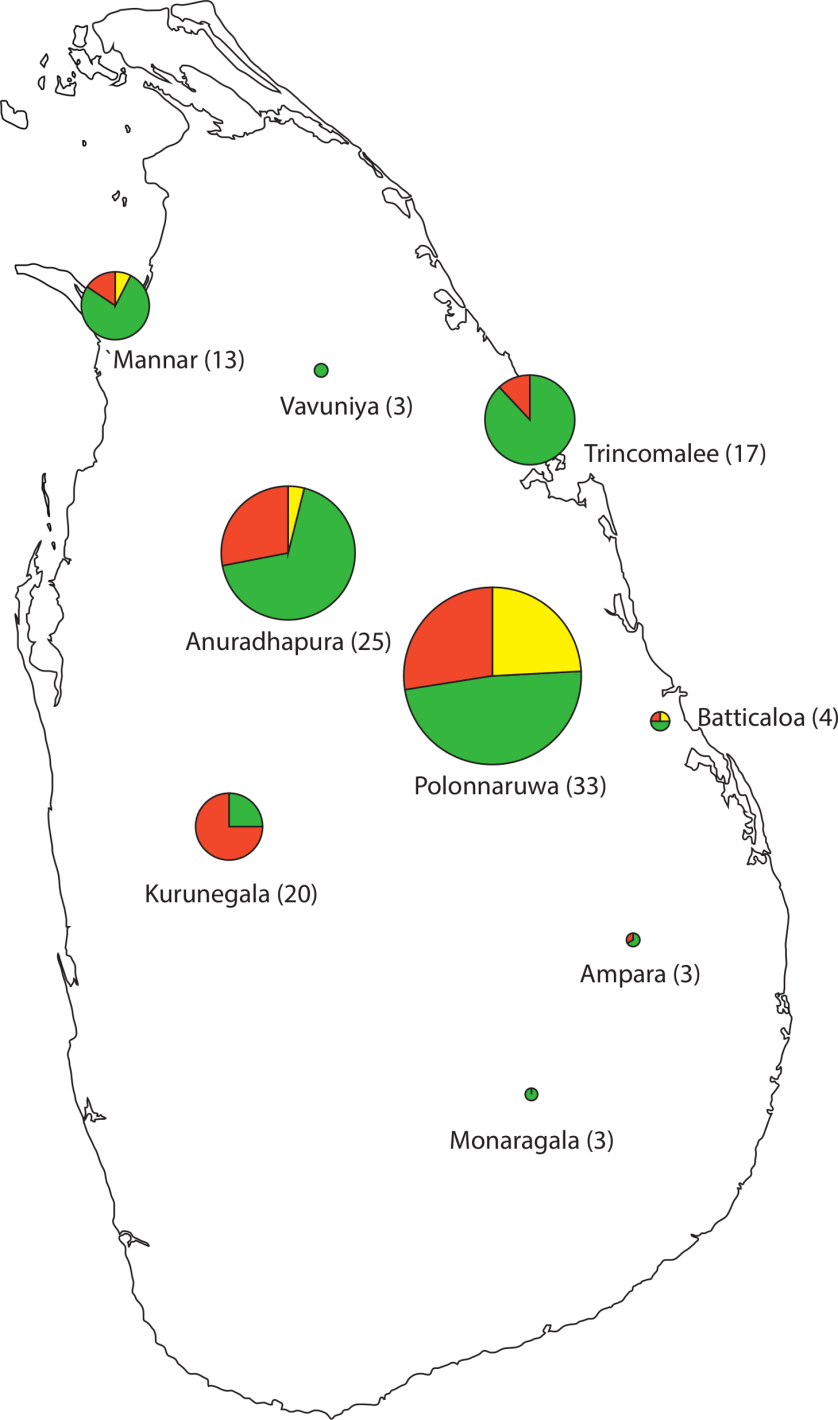
Genetic diversity of the *Plasmodium vivax mdr1* gene in *P*. *vivax* samples collected at 9 malaria endemic districts of Sri Lanka. Three different genotypes were detected in the samples from Sri Lanka named MYF(yellow), MYL(green) and MFL(red). MYF (single mutant, T958M), MYL (double mutant, T958M and F1076L) and MFL (triple mutant). The number of positive samples from each district is mentioned in brackets.

District level analysis was possible for Sri Lanka (Fig 3). In Sri Lanka, samples were collected in 9 districts, and despite the small sample size per district, the distribution of alleles was similar at district level when compared to the pooled sample set, with a dominating MYL haplotype, followed by MFL and MYF. The exception was Kurunegala district where the MFL haplotype was most common (n = 15), followed by the MYL haplotype (n = 5).

### Analysis of the four microsatellites

Four MS from *Pvmdr1* flanking genomic regions were genotyped in the 267 samples although these were amplified with varying successes; for m9.5 (n = 190), m10.1 (n = 196), m10.4 (n = 229) and m43.1 (n = 181) alleles (Table 2). The m10.1 locus was the most polymorphic with 26 alleles identified among 196 samples (H_e_ = 0.90), while m9.5 had 4 alleles, m10.4 had 13 and m43.1 had 3 different alleles (Table 2, and S1 Table). The number of mixed samples (those containing more than one allele) detected using each locus differed; only 1% were mixed at the m9.5 locus, while 12% were mixed at the m43.3 locus. When combining all 5 loci, 26% were mixed among 125 samples (Table 2).

The m10.1 locus had a mono-A-repeat as well as an AT dinucleotide repeat, and this was reflected in the high number of different alleles (H_e_ = 0.90). Ecuador was an exception, only possessing 4 different m10.1 alleles among the 16 positive samples (H_e_ = 0.68). Allele size variation is shown in S1 Table, the highest number of observed MS alleles were generally of intermediate size.

**Fig 4:**
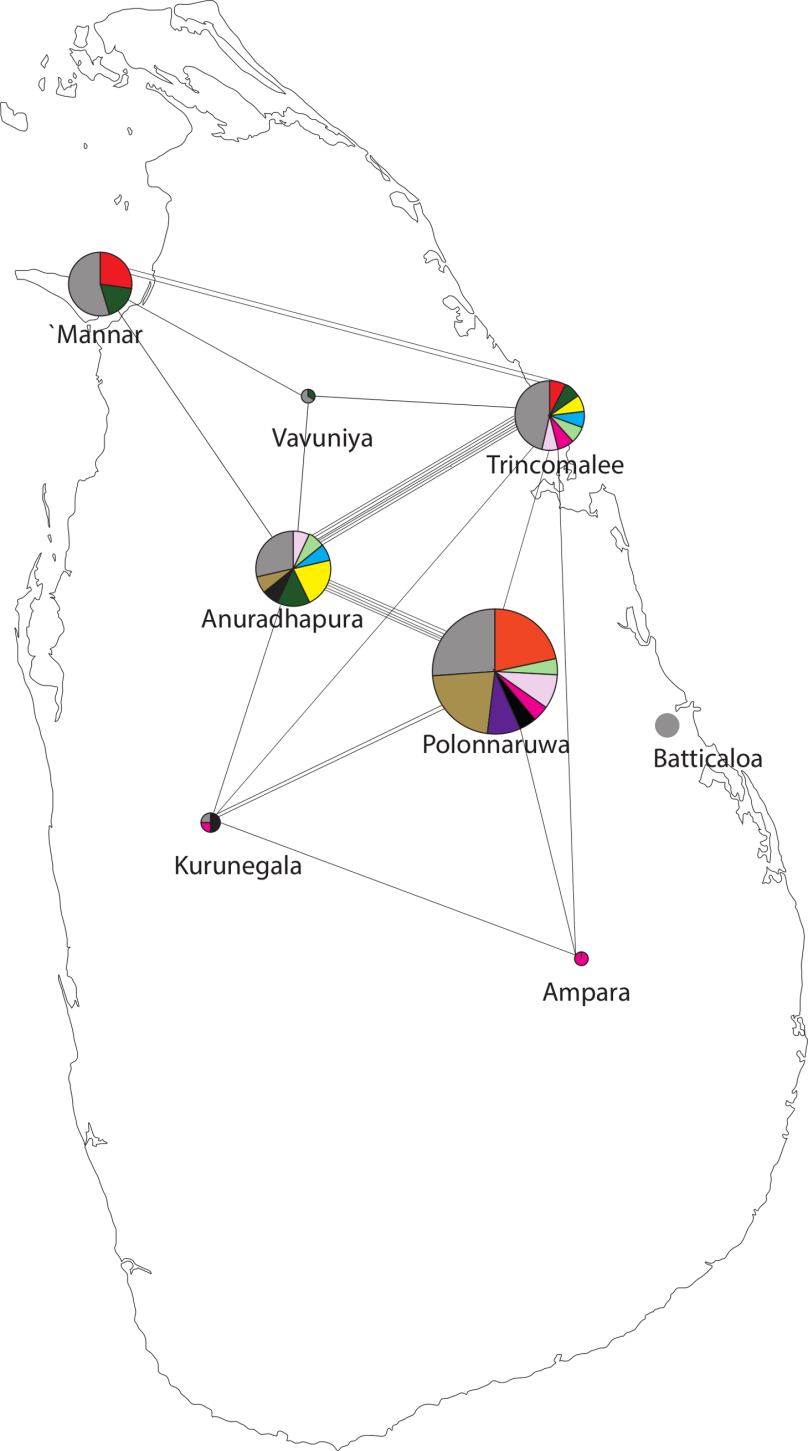
*Plasmodium vivax mdr1* combined with flanking microsatellite variation in *P*. *vivax* samples collected at 9 malaria endemic districts of Sri Lanka. The size of the pie charts reflects the sample size, while the segments illustrate the frequency of combined *Pvmdr* SNP+ microsatellite haplotypes. Grey color segment indicates the proportion of the sample which consisted of unique 'singleton' haplotypes while the other colors indicate the proportion of each repeated haplotype. The black lines indicate sharing of a specific haplotype between two sites and multiple lines indicate the number of haplotypes shared between given sites.

The combination of all 5 loci resulted in 57 different 5-loci haplotypes among the monoclonal samples (Table 3) and 78 when majority alleles in the mixed genotype samples were included. All 5-locus haplotypes differed from the CQS wild-type Sal-1 haplotype. The most commonly observed 5-locus haplotype was detected in Ecuador (n = 6, haplotype number 8 in Table 3). The other frequently observed 5-loci haplotypes were all from Sri Lanka. The distribution of the *Pvmdr1* 5- locus haplotypes among the sub-populations sampled in Sri Lanka is shown in Fig 4. A large number of haplotypes occurred only once and these are indicated by grey segments in the pie charts. Haplotypes which occurred multiple times are indicated each by a different colour. The sample collections with the greatest degree of haplotype sharing were from Trincomalee and Anuradhapura (6 haplotypes) and Anuradhapura and Polonaruwa (5 haplotypes of which only 1 was found in Trincomalee). Other sites appear more isolated, for instance all the haplotypes found in the district of Batticaloa were unique.

Unfortunately, the small amount of DNA-solution available from Sudan and São Tomé prevented reanalysis of these samples, and no 5-loci haplotype from Sao Tomé could be created.

We tested for LD, between *Pvmdr1* alleles and flanking MS alleles. Significant associations are shown in S2 Table. Strong linkage associations were seen in Ecuador where MS alleles occurred in association with the MYF single mutant allele and also with the wildtype allele TYF. Other population level LD associations were observed in Sri Lanka, and Nepal. In each case different MS alleles were associated with the *Pvmdr1* allele. When the 4 MS loci were combined, significant LD between certain 4-loci haplotypes and either the TYF, MYF or MFL haplotypes was found in Ecuador and Sri Lanka, (S2 Table). When samples from all sites were pooled the LD analysis found significant associations of MS alleles with TYF and MFL which are likely attributable to admixture.

## Discussion

The aim of this study was to characterise SNPs in the *Pvmdr1* gene, to examine whether the putative CQR mutations have one, few or many origins, and to determine whether there has been geographical spread of certain *Pvmdr1* haplotypes. SNPs were found at three codons, T958M, Y976F and F1076L among 267 samples from Pakistan, Nepal, Sri Lanka, Ecuador, Sudan and São Tomé. Polymorphism in the last two codons has been described in multiple studies [11;14;15;18;19;22;23], but the high prevalence of 958M which we observed (206/211, 97.6%) was surprising since this SNP has only been mentioned in two earlier studies; in Madagascar (with a 100% fixation of the 958M) [18] and in a few samples from Indonesia and Brazil [39]. Besides these studies, all others either report the presence of the wild-type T958 allele, or do not mention the locus [11;20–23]. Since the 958M mutation was present in countries with both high and low level of reported CQR over a wide time span, we hypothesize that the T958M is an allelic variant of the wildtype and most likely not associated with CQR. In the present study the T958 wild-type was only detected in Ecuador. It is also seen in the Sal-1 reference sample which originates from El Salvador, so it is possible this allele might be a characteristic of American samples while the 958M is characteristic of Asia and Africa.

Rare mutations, F979S (TTT→TCT), M980V (ATG→GTG) and S1080N (AGT→AAT) were found in two samples from the Jhapa district of Nepal, both possessing the MYL double mutant *Pvmdr1* haplotype (Table 3); One of these samples was mutated at codon 1080 while the other was mutated at codon 979 and codon 980. These results have been previously published by Ranjitkar in 2011 [35]. Thus, in total only five *Pvmdr1* SNPs were detected which was an unexpected result. Orjuela-Sanchez *et al*. (2009) [23] reported up to 24 *Pvmdr1* mutations in a study of only 7 samples from Brazil, while Barnadas *et al*. (2008) [18] reported 21 mutations among 105 samples from Madagascar. However, both studies amplified longer fragments of the *Pvmdr1* gene than the present study, which might be a part of the explanation.

Genotyping of microsatellites flanking the *Pvmdr1* gene revealed high levels of diversity around single, double and triple mutant alleles. There were too few wildtype TYF alleles to meaningfully compare the MS heterozygosity surrounding wildtype and mutant alleles for evidence of selective sweeps on the mutant alleles. However our finding that all 3 wildtype TYF alleles were flanked by an identical microsatellite haplotype would not support the view that reduced diversity among microsatellite haplotypes is attributable to selective sweeps, but rather suggests a tendency to clonal population structure in some populations.

The evidence for association of MS alleles with particular mutations was patchy. TYF and MYF *Pvmdr1* haplotypes occurred together with the “201” m9.5 allele, while the “204” allele at m9.5 was more commonly seen with MFL and MYL. No obvious pattern of distribution was seen for the other 3 MS, suggesting this association was caused by greater representation of certain mutant alleles in particular geographic localities rather than a selective sweep.

The combination of the 5 *Pvmdr1* loci into a 5-loci haplotype revealed 57 different haplotypes among the 125 samples positive at all loci, many of them unique. Country-wise, Nepal was the most diverse, when analysed by locus and for the combined 5-loci haplotype, whereas Ecuador was more conserved. The diminished diversity within the Ecuadorian samples is consistent with the general finding of little diversity amongst *P*. *vivax* samples from the Americas, although this is not a hard and fast rule [40]. Just 10 samples of African origin were available for this study but both double and triple *Pvmdr1* mutant alleles were present, and their microsatellite fingerprint was distinct from that associated with the same alleles in Asia. Likewise, our South American sample from Ecuador (n = 17) was distinct from the other populations being less diverse, and unique in having the wild-type *Pvmdr1* allele.

Studies of *P*. *falciparum* have revealed a contrasting pattern of resistance evolution in which relatively few resistance mutants emerge but then become globally disseminated. The pattern is consistent for both CQR [26;41] and high levels of resistance to SP [24;27;42–44]. Hawkins *et al*. [28;29] analysed the origin and dissemination of SPR in *P*. *vivax* by analysing SNPs in and surrounding the *Pvdhfr* and *Pvdhps* genes. They concluded that the genes are considerably more diverse than seen in *P*. *falciparum* [28;29] and that highly pyrimethamine-resistant *Pvdhfr* alleles arose three times in Thailand, Indonesia and PNG/Vanuatu, and that sulfadoxine resistance associated SNPs had evolved independently on multiple occasions. This is consistent with our findings on Pvmdr and may be explained by comparisons of total genomic diversity among *P*. *vivax* and *P*. *falciparum* isolates. A study by Neafsey *et al*. [45] reported twice as much SNP diversity, significantly higher MS diversity and a far deeper divergence among *P*. *vivax* geographic isolates than among a comparable set of *P*. *falciparum* isolates. The higher level of diversity in *P*. *vivax* can explain the multiple origin pattern of resistance emergence in of *Pvmdr1*, *Pvdhfr* and *Pvdhps*.

Our findings can indicate three things. First, *Pvmdr1* mutant alleles have developed on multiple haplotype backgrounds by convergent evolution in Pakistan, Nepal, Sri Lanka, Ecuador, Sao Tomé and Sudan. Second, assuming that *Pvmdr1* is a reliable CQR marker, there is little evidence that the variation around mutant haplotypes has been subject to a selective sweep, (the result of positive natural selection causing diminished diversity in sequences flanking the selected marker). Third, the historical pattern of drug resistance emergence in *P*. *falciparum* is not repeated in *P*. *vivax*. The time-delay of around 30 years between initial reports of CQR in the two malaria species, and the two different treatment regimens (mono-in *P*. *falciparum* and usually combined CQ/primaquine treatment in *P*. *vivax*) might explain this, and may suggest that it is just a matter of time before the effect of selection of the markers can be seen since high grade treatment failure has not yet been reported in any of the sites sampled in this study. *P*. *vivax* is generally a chronic disease with low parasitaemia causing mild symptoms compared to *P*. *falciparum*, therefore there is less selective drug pressure on the parasite. Furthermore, the fast gametocytogenesis in *P*. *vivax* enables uptake of gametes by vectors before clinical symptoms arise and antimalarial treatment is initiated which balances the spread of sensitive and resistant *P*. *vivax* parasites.

Equally, these differences may lie with the biology and transmission dynamics of the two species. Our district level analysis of *Pvmdr* and linked MS variation in Sri Lanka found evidence of exchange of genotypes between districts which may or may not be linked to their resistance phenotype. Latent *P*. *vivax* infections cause relapses of the disease, which may increase the possibility of migration of parasites within the human host while the broader temperature tolerance by *P*. *vivax* parasites compared to *P*. *falciparum* might increase the likelihood of gene flow between sites with varying temperature or microclimate.

Finally CQR may be a complex trait including other genes in addition to *Pvmdr1* and future research will hopefully illuminate the genomic-level change underpinning changes in CQ sensitivity. Meanwhile, monitoring and research of CQR is of highest importance for the public health in the afflicted areas.

## Supporting Information

S1 Table The number of detected alleles per collection site and locus; the*Pvmdr1* gene and the four microsatellites m9.5, 10.1, 10.4, and 43.1. The *Pvmdr1* alleles are named TYF (wild-type allele), MYF, MYL and MFL, corresponding to the codes of the amino acids encoded by the polymorphic codons 958, 976 and 1076, respectively.
(DOCX)

S2 Table Linkage disequilibrium analysis between *Pvmdr1* alleles and alleles of flanking microsatellites. Significant LD (D*'* = 1, P < 0.05) between *Pvmdr1* alleles (TYF, MTF, MYL, and MFL) and alleles of each of three flanking microsatellites (MS); m9.5, m10.1, and m10.4. Significant LD was not found between the Pvmdr1 alleles and m43.1 alleles. LD calculations were carried out for the total pooled sample (T) and for individual populations in Pakistan, Nepal, Sri Lanka, Ecuador, Sao Tomé and Sudan. Significant LD could only be detected in Ecuador (E), Sri Lanka (SL) and Nepal (N) and in the total sample set (T). In brackets is the number of alleles in complete LD detected per study site. Excluded in the table are haplotypes with frequencies >1 and mixed infection samples. Pairwise significance levels are as follows: “***”significance at the 0.1% nominal level, “**” significance at the 1% nominal level and “*” significance at the 5% nominal level.
(DOCX)
