# Supplementary material for: Multiple Origins of Mutations in the mdr1 Gene—A Putative Marker of Chloroquine Resistance in P. vivax
Source: PLoS Negl Trop Dis. 2015 Nov 5;9(11):e0004196. doi: 10.1371/journal.pntd.0004196 (PMC4634971; doi:10.1371/journal.pntd.0004196)
Supplement: S1 Table — The Pvmdr1 alleles are named TYF (wild-type allele), MYF, MYL and MFL, corresponding to the codes of the amino acids encoded by the polymorphic codons 958, 976 and 1076, respectively. (DOCX) [file pntd.0004196.s001.docx]

| Locus | **Alleles** | **Pakistan/Afghanistan** | **Nepal** | **Sri Lanka** | **Ecuador** | **Sao Tomé** | **Sudan** |
| --- | --- | --- | --- | --- | --- | --- | --- |
| *Pvmdr1* | TYF |  |  |  | 5 |  |  |
|  | MYF |  | 6 | 10 | 12 |  |  |
|  | MYL | 24 | 35 | 72 |  |  | 2 |
|  | MFL |  | 2 | 38 |  | 3 | 2 |
|  | *N* (*Pvmdr1* alleles) | 24 (1) | 43 (3) | 120 (3) | 17 (2) | 3 (1) | 4 (2) |
| m9.5 | 199 |  | 1 |  |  |  |  |
|  | 201 | 1 | 7 | 25 | 17 |  |  |
|  | 204 | 19 | 20 | 90 |  | 2 | 3 |
|  | 207 |  | 4 |  |  |  | 1 |
|  | *N* (m9.5 alleles) | 20 (2) | 32 (4) | 115 (2) | 17 (1) | 2 (1) | 4 (2) |
| m10.1 | 266 |  | 3 |  | 5 |  |  |
|  | 268 |  |  |  |  | 1 |  |
|  | 271 |  | 1 |  |  |  |  |
|  | 277 |  | 1 |  |  |  |  |
|  | 280 | 4 | 2 |  |  |  |  |
|  | 282 |  |  | 6 |  |  |  |
|  | 283 | 2 |  | 26 |  |  |  |
|  | 286 |  | 1 |  |  |  |  |
|  | 287 | 5 |  |  |  |  |  |
|  | 288 |  |  | 1 |  |  |  |
|  | 289 |  | 6 | 36 |  |  |  |
|  | 290 | 1 | 1 |  |  |  |  |
|  | 291 | 1 |  | 8 |  |  |  |
|  | 293 |  | 3 |  |  |  | 3 |
|  | 297 |  |  | 12 |  | 1 |  |
|  | 298 |  | 1 | 2 |  |  |  |
|  | 299 |  | 3 | 21 |  |  |  |
|  | 300 |  | 1 | 2 | 8 |  |  |
|  | 301 | 1 |  |  | 2 |  |  |
|  | 303 | 2 |  | 1 | 1 |  | 1 |
|  | 305 |  | 3 | 1 |  |  |  |
|  | 311 |  |  | 3 |  |  |  |
|  | 308 |  | 1 |  |  |  |  |
|  | 313 | 1 |  |  |  |  |  |
|  | 315 |  | 1 |  |  |  |  |
|  | 322 |  | 1 |  |  |  |  |
|  | *N* (10.1 alleles) | 17 (8) | 29 (15) | 119 (12) | 16 (4) | 2 (2) | 4 (2) |
| m10.4 | 230 |  | 3 | 1 |  | 2 |  |
|  | 233 |  | 2 |  |  |  |  |
|  | 244 | 3 |  | 1 |  |  |  |
|  | 247 |  | 2 |  |  |  |  |
|  | 252 | 4 | 7 | 24 | 7 |  |  |
|  | 255 | 4 | 3 | 24 |  |  |  |
|  | 259 | 11 | 14 | 25 |  |  |  |
|  | 261 | 1 | 1 |  | 3 |  |  |
|  | 266 | 16 | 11 | 22 | 6 |  |  |
|  | 268 |  |  |  |  |  |  |
|  | 270 |  |  | 24 |  |  | 1 |
|  | 276 |  | 1 | 1 |  |  | 2 |
|  | 283 |  | 1 |  |  |  |  |
|  | 293 |  |  |  |  |  |  |
|  | 308 |  |  |  |  |  | 2 |
|  | *N* (m10.4 alleles) | 39 (6) | 45 (10) | 122 (8) | 16 (3) | 2 (1) | 5 (3) |
| m43.1 | 419 | 20 | 26 | 61 | 11 |  | 2 |
|  | 437 | 9 | 4 | 36 | 6 |  | 1 |
|  | 472 |  | 4 |  |  | 1 |  |
|  | *N* (m43.1 alleles) | 29 (2) | 34 (3) | 97 (2) | 17 (2) | 1 (1) | 3 (2) |
