# Supplementary material for: Multiple Origins of Mutations in the mdr1 Gene—A Putative Marker of Chloroquine Resistance in P. vivax
Source: PLoS Negl Trop Dis. 2015 Nov 5;9(11):e0004196. doi: 10.1371/journal.pntd.0004196 (PMC4634971; doi:10.1371/journal.pntd.0004196)
Supplement: S2 Table — Significant LD (D' = 1, P < 0.05) between Pvmdr1 alleles (TYF, MTF, MYL, and MFL) and alleles of each of three flanking microsatellites (MS); m9.5, m10.1, and m10.4. Significant LD was not found between the Pvmdr1 alleles and m43.1 alleles. LD calculations were carried out for the total pooled sample (T) and for individual populations in Pakistan, Nepal, Sri Lanka, Ecuador, Sao Tomé and Sudan. Significant LD could only be detected in Ecuador (E), Sri Lanka (SL) and Nepal (N) and in the total sample set (T). In brackets is the number of alleles in complete LD detected per study site. Excluded in the table are haplotypes with frequencies >1 and mixed infection samples. Pairwise significance levels are as follows: “***”significance at the 0.1% nominal level, “**” significance at the 1% nominal level and “*” significance at the 5% nominal level. (DOCX) [file pntd.0004196.s002.docx]

| MS | Allele | TYF | MYF | MYL | MFL |
| --- | --- | --- | --- | --- | --- |
| m9.5 | 201 | T (5)*** |  |  |  |
| m10.1 | 266 | E (4)***, T (4)*** |  |  |  |
|  | 291 |  |  |  | SL (6)***, T (6)*** |
|  | 297 |  |  | SL (9)* |  |
|  | 298 |  | N (2)* |  |  |
|  | 300 |  | E (8)* |  |  |
| m10.4 | 230 |  |  |  | T (2)** |
|  | 259 |  |  | N (13)* |  |
